# Relations of Psychosocial Factors and Cortisol with Periodontal and Bacterial Parameters: A Prospective Clinical Study in 30 Patients with Periodontitis Before and After Non-Surgical Treatment

**DOI:** 10.3390/ijerph17207651

**Published:** 2020-10-20

**Authors:** Marie Dubar, Isabelle Clerc-Urmès, Cédric Baumann, Céline Clément, Corentine Alauzet, Catherine Bisson

**Affiliations:** 1 Department of Periodontology, School of Dentistry, Lille University Hospital, 59000 Lille, France; 2 Stress Immunity Pathogens Unit (SIMPA), EA 7300, University of Lorraine, F-54000 Nancy, France; c.alauzet@chru-nancy.fr (C.A.); catherine.bisson@univ-lorraine.fr (C.B.); 3 Department of Methodology, Promotion and Investigation, UMDS, University Hospital of Nancy, 54500 Vandoeuvre-lès-Nancy, France; I.CLERCURMES@chru-nancy.fr (I.C.-U.); c.baumann@chru-nancy.fr (C.B.); 4 CHRU Nancy, Department of Public Health Dentistry, University Hospital, 54000 Nancy, France; celine.clement@univ-lorraine.fr; 5 “Interpsy” Laboratory, University of Lorraine, EA 4432, CEDEX 54015 Nancy, France; 6 «Health Systemic Process» Laboratory, University Lyon 1, EA 4129, 69008 Lyon, France; 7 CHRU Nancy, Microbiology Department, University Hospital, F-54000 Nancy, France; 8 Department of Periodontology, Nancy University Hospital, Lorraine University, 54500 Vandoeuvre-lès-Nancy, France

**Keywords:** periodontitis, stress, anxiety, cortisol, periodontal parameters, periodontal therapy, periodontal bacteria

## Abstract

(1) Background: The progression of periodontitis, induced by polymicrobial dysbiosis, can be modified by systemic or environmental factors such as stress or anxiety affecting host response. The purpose of this study is to evaluate the potential associations between psychosocial factors scores or salivary cortisol levels with clinical periodontal parameters and bacterial environment in patients with periodontitis; (2) Methods: Subgingival microbiota was collected in two pathological and one healthy sites from thirty diseased patients (before/after scaling and root planing (SRP)) and from one healthy site from thirty control patients. Usual clinical periodontal parameters were recorded, and a saliva sample was harvested. Patients completed stress and anxiety self-assessment questionnaires. Cortisol concentrations were determined by ELISA and bacteria were identified by PCR; (3) Results: No correlation between salivary cortisol and the stress-anxiety self-declared was found (*p* > 0.05), but high concentrations of this molecule were associated positively and linearly with periodontal pocket depth (*p* = 0.04). It appeared that certain psychosocial stressors are associated with a modulation of the bacterial colonization of pockets of diseased group (before/after SRP), notably concerning *Tannerella forsythia* (*p* = 0.02), *Porphyromonas gingivalis* (*p* = 0.03), *Fusobacterium nucleatum* (*p* = 0.049) and *Campylobacter rectus* (*p* = 0.01). (4) Conclusion: This study reveals associations between bacteria colonization and psychosocial parameters in periodontitis that needs to be further investigated.

## 1. Introduction

Periodontitis is a multifactorial disease from polymicrobial origin composed of inflammatory lesions of the superficial (gingiva) and deep periodontal tissues (alveolar bone, periodontal ligament and cementum) which can lead to teeth loss in the absence of therapy [1]. This disease is the 6th most widespread disease in the world. Because of its considerable economic impact [2] and its numerous interrelationships with certain systemic diseases (e.g., diabetes, cardiovascular disease, Alzheimer’s disease) [3,4], periodontitis has become a public health concern. A complete understanding of the etiopathogenesis of periodontitis as well as the improvement of their treatment, are required and need to be further studied.

Periodontopathogens, that could be present in the biofilm, are recognized as the causal agents of periodontitis and participate to the oral ecosystem imbalance. They include red Socransky’s complex bacteria (*Porphyromonas gingivalis*, *Tannerella forsythia*, *Treponema denticola*) as well as *Aggregatibacter actinomycetemcomitans* and to a lesser extent, orange complex bacteria (e.g., *Fusobacterium nucleatum*, *Campylobacter rectus*, *Prevotella intermedia*) which all produce a wide variety of virulence factors enabling them: (i) to colonize, invade and destroy the gingival tissues to recover nutrients (peptides/proteins) and (ii) to resist host defense mechanisms (inhibition of phagocytosis, lysis of immune cells and destruction of complement molecules) [5,6,7]. The immuno-inflammatory responses induced by their presence in the periodontal microbiota are mediated by cytokines and chemokines that interact as a network in the periodontium [8].

The onset and development of periodontitis can be influenced by many factors, modifiable or non-modifiable, acquired or innate, known as risk or modifying factors. Based on current scientific knowledge, certain conditions such as diabetes and smoking have been identified as risk factors for periodontitis [9]. However, stress or anxiety, universal physiological reaction, are increasingly identified as independent factors that can directly compromise periodontal disease through various biological or behavioural mechanisms when they become excessive and chronic [10]. Indeed, observational results from clinical studies show that stress and anxiety leading to depression can increase unhealthy behaviours: (i) poor oral hygiene, (ii) increase in tobacco or alcohol consumption and (iii) poor nutrition [11,12]. Furthermore, by activating the hypothalamic-pituitary-adrenal axis and the sympathetic nervous system through the production of cortisol and catecholamines, psychosocial stress disrupts the balance between pro-inflammatory and anti-inflammatory responses. This disruption results, among other things, in an immunosuppressive action thanks to the presence of glucocorticoid receptors on the surface of several immune cells (e.g., inhibition of T-lymphocytes formation and suppression of the function of natural killer cells or macrophages) [13]. In addition, cortisol molecules can also disturb the metabolism of fats, proteins and glucose and thus increase the sensitivity of periodontal tissues to periodontopathogens [14].

Numerous reports suggest that microorganisms would have had large time during evolution of the life to be in contact with a wide spectrum of endocrine hormones produced by animals and plants, and therefore to develop the ability to synthetize and recognize them [15]. Following this discovery, a new concept has evolved known as the “microbial endocrinology” in which a bidirectional interaction between microorganisms and human neuroendocrine factors has been observed. Recently several studies have analyzed the periodontopathogens ability to be affected by stress hormones according to the emergence of this concept [15,16]. For example, stress hormones such as catecholamines can alter the course and outcome of certain intestinal infections by altering the bacteria motility or growth, the *quorum sensing*, the virulence and the biofilm formation of intestinal species such as *Escherichia coli* or *Pseudomonas aeruginosa*, in animal models [17]. The effects of these hormones on the microorganisms provided an interesting explanation of stress-induced alterations observed in the infectious process and led researchers to explore these pathways in periodontitis. In vitro studies have shown that, addition of norepinephrine, induced *P. gingivalis* and *A. actinomycetemcomitans* growth decrease while a growth increase was described for *Eikenella corrodens* [18]. It was also observed that cortisol could directly reduce the metabolic activity of *F. nucleatum* at very high doses as well as its vitality and that of *P. intermedia*, *E. corrodens* and *T. forsythia* [19]. To date, few studies have clinically analyzed the impacts of stress and/or anxiety on the presence of periodontopathogens and abundance of total flora in periodontal diseases.

The aim of this clinical study was to determine the interrelationships between salivary cortisol concentrations, self-reported stress and anxiety levels, periodontal clinical parameters and presence of crevicular periodontopathogens in patients with periodontitis before and after scaling and root planing (SRP). Our study hypotheses were that: (i) high cortisol concentrations are related to high stress/anxiety scores (ii) with more anxious and stressed patients in periodontitis group than in control group and (iii) high cortisol levels, or stressed and anxious periodontitis patients are associated with the worst periodontal parameters and present modulation of periodontopathogens detection compared to non-stressed/non-anxious periodontitis patients or with low cortisol levels before and after SRP.

## 2. Materials and Methods

### 2.1. Patients

Patients referred to the periodontology department of Nancy University Hospital were invited to participate in this preliminary monocentric clinical study between April 2016 and October 2017. 30 adult patients (≥18 years old): (i) with moderate to severe chronic periodontitis [20] corresponding to stage II-IV/grade A-C of the new classification of periodontal conditions and diseases [21]), (ii) with at least two periodontal pockets (probing depth ≥5 mm) and one healthy site (probing depth ≤3 mm), (iii) without diseases (such as diabetes) or antimicrobials, anti-inflammatory or other drugs affecting immune status or bacterial ecosystem (such as channel blockers and anti-epileptics), (iv) with current non-pregnancy and (v) without a periodontal therapy in the six previous months, were considered candidates for the study and have formed the diseased group.

Thirty other adult patients receiving dental check-ups or scaling with: (i) no periodontal disease (ii) in good health (no systemic disease) and with (iii) current non-pregnancy were proposed to participate to the study and formed the control group.

**Scheme 1:**
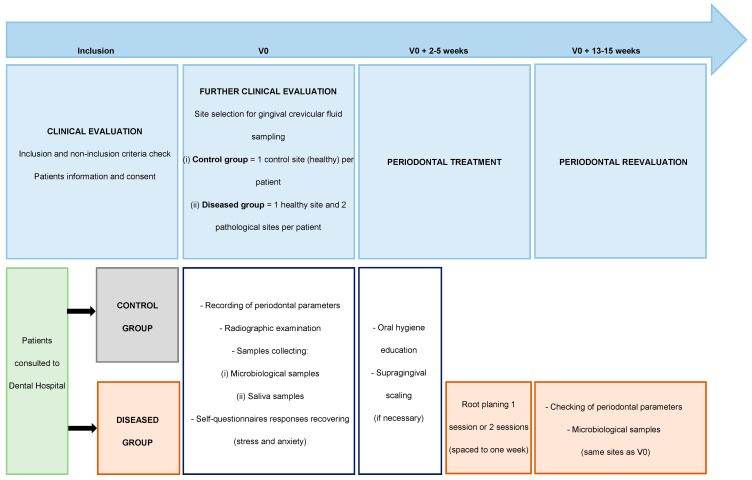
Study design.

The recruitment of patients was prospective and extensive, based on clinical and radiographic analysis for both groups (diseased and control). This case-control study with intervention in cases, provided in Scheme 1, was independently approved by the Ethical Committee of Nancy University Hospital (CPP/16/03/04 decision number) and received NCT02873949 clinicaltrials.com number. Informed and written consent was provided from each participant before participation.

### 2.2. Clinical Evaluation

All patients were examined by the same calibrated operator. The usual periodontal clinical parameters were first analyzed to identify the patients who met the criteria for inclusion and non-inclusion. In a second time, radiographic examination and the following periodontal clinical parameters were meticulously recorded for all teeth of patients with periodontitis, among them the two deepest pathological and one healthy sites were selected for bacterial samples: (i) periodontal pocket depth (PPD) or probing depth, (ii) level of clinical attachment (CAL) using a William’s graduated probe, (iii) presence of bleeding on probing (BoP), (iv) presence of tooth mobility, (v)) plaque index (PlI) [22], and (vi) gingival index (GI) [23]. The same protocol (periodontal parameters recording and bacterial sample) was used for one healthy site called control site in control patients.

### 2.3. Salivary Sampling

A saliva sample was taken from each patient in both groups using the Salivette^®^ device (Sarstedt, Marnay, France) in accordance with the manufacturer’s instructions. This device was given to the patients on the day of inclusion for collection in the morning of their next visit when they woke up. Briefly, before eating, drinking, smoking or practicing oral hygiene (dental brushing, interdental, mouthwash), patients removed and placed the swab in their mouths without touching it for 2–3 min until it was soaked. The swab was then replaced in its tube and returned to the department during the day with temporary storage in the refrigerator. The received tube was immediately centrifuged at 1000× *g* for 1 min and frozen at −80 °C until analysis.

### 2.4. Microbiological Sampling

The selected teeth (two pathological and one healthy sites in diseased group and one control site in control group) were cleaned supragingivally with a cotton roll and then isolated from saliva. Microbiological sampling was carried out through the gingival crevicular fluid (GCF) collection using two sterile paper points of diameter 40, per selected site, inserted 30 s into the sulcus or periodontal pocket. The collected paper points of each site were then placed in separate empty encoded sterile Eppendorf tubes and stored at −80 °C until analysis.

### 2.5. Psychosocial Stress and Anxiety Measurements

Two questionnaires were chosen to assess the stress and anxiety of diseased or control patients: (i) the State Anxiety Inventory (STAI-Y), which is composed of two scales, the State Anxiety Scale (STAI-YA) and the Trait Anxiety Scale (STAI-YB) [24,25] and (ii) Cohen’s Perceived Stress Scale (PSS) [26]. These self-administered questionnaires were completed by patients at the end of the Inclusion Day consultation. These three questionnaires were chosen because their psychometric properties have been confirmed in the literature [27,28].

Briefly, the STAI-YA scale assesses the person’s anxiety at the time of filling in a potentially anxiety-provoking situation that may be dental care. The STAI-YB scale assesses whether the patient has an anxious personality or whether his daily anxiety may be pathological. Each item is scored from 1 to 4 points and high scores indicate high levels of anxiety. Each patient was divided into 3 categories: non-anxious (STAI-YA: men<37 and women<42; STAI-YB: men<39 and women<47), anxious and very anxious (STAI-YA: men>48 and women>55; STAI-YB: men>51 and women>61). The PSS scale measures each patient’s perception of stress in the last month and whether they can manage it. It is an inventory of 10 questions, each rated from 1 to 5 points. High scores indicate high levels of stress. Each patient was divided into 3 categories: unstressed (<21), managed stressed, very stressed (>27).

### 2.6. Cortisol Quantification

Levels of salivary cortisol concentrations were determined from saliva sampling using commercially available specific enzyme-linked immunosorbent assay (ELISA) kits (R&D Systems, Minneapolis, MN, USA) according to manufacturer’s instructions. Absorbance at 450 nm was measured using a microplate reader (Multiskan™, Thermo Fisher Scientific, Waltham, MA, USA) with a wavelength correction set at 570 nm. The detection limit of this test is 0.156 ng/mL (0.429 nmol/L).

### 2.7. Bacteria Detection and Quantification

From the sterile paper points used for microbiological sampling, bacterial DNA was extracted and purified with the QIAamp DNA mini kit^®^. Thus, 400 µL of AL buffer solution was added to the tubes and incubated at 56 °C for 30 min with 20 µL of proteinase K (lysis step). Several washes were then performed and finally the DNA was resuspended in 75 µL of elution buffer. These steps followed the manufacturer’s instructions. The quantity and purity of the extracted DNA was evaluated with a Nanodrop™2000 spectrometer (Thermo Fisher Scientific, Waltham, MA, USA).

On the one hand, the total bacterial flora was quantified using real-time PCR by the Institut Clinident SAS (Aix-en-Provence, France). Primers targeting 16S rDNAs were used as previously described [29] with a minimum detection threshold of 1.10^4^ and a minimum quantification threshold of 2 × 10^4^ 16S rDNAs copies.

On the other hand, the extracted DNA was then amplified and hybridized with the MicroIDent^®^Plus 11 kit (Hain LifeScience, Nehren, Germany) to identify 11 periodontopathogens belonging to different Socransky complexes with a minimum threshold of 10^4^ bacteria in the sample: *P. gingivalis*, *T. denticola*, *T. forsythia* (red complex bacteria); *P. intermedia*, *Parvimonas micra*, *Eubacterium nodatum*, *F. nucleatum/periodonticum*, *C. rectus* (five orange complex bacteria); *E. corrodens* and *Capnocytophaga* spp. (two green complex bacteria) as well as *A. actinomycetemcomitans* serotype b (with a specific minimum threshold of 10^3^ bacteria for this species). Briefly, 5 µL of extracted DNA was added to 45 µL of reaction mixture containing specific primers, nucleotides, buffer and Taq polymerase as described above [30]. A PCR cycle with a denaturation step (95 °C, 5 min) then 10 cycles (95 °C, 30 S-58 °C, 2 min) then 20 cycles (95 °C, 25 s-53 °C, 40 s-70 °C, 40 s) and a final extension step (70 °C, 8 min).

Amplification was followed by a manual combined reverse hybridization where the biotin-labelled amplicons were denatured using a hybridization buffer for 30 min at 45 °C in the Twincubator^®^ (Biocentric, Bandol, France) platform. In order to remove unbound DNA, a washing step is performed followed by the addition of streptavidin-conjugated alkaline phosphatase and after a final washing step by the addition of substrate concentrate containing dimethyl sulfoxide. The manufacturer’s instructions have been followed for amplification and hybridization. The strips were interpreted by two independent evaluators. The presence of a colorimetric band indicated the presence of the bacteria in the sample. The accuracy of this test has been previously evaluated in the literature [31,32].

### 2.8. Periodontal Therapy

All patients in the study received oral hygiene instructions. Briefly, in order to achieve optimal plaque control, all patients have received oral hygiene instructions thanks to a demonstration of technique and duration of tooth brushing with their own equipment. Advice on the most suitable material for interdental cleaning (choice of interdental brushes and/or dental floss), advice on the maintenance and renewal of equipment for toothbrushing were also given. Diseased patients then received non-surgical periodontal treatment including scaling and root planing with manual curettes and ultrasound of all periodontal lesions. The treatment was performed in one or two sessions spaced one week apart by two experienced periodontists in the department. A periodontal reassessment, 60–75 days after the last treatment session, was performed by the same operator, and the same different periodontal parameters as those before initial treatment were collected without having access to these parameters recorded during the initial consultation. Periodontal clinical parameters and microbiological samples from the same selected sites were collected before and after treatment.

### 2.9. Statistical Analysis

Categorical data were presented as numbers (%); continuous data were described as median [Minimum-Maximum]. Comparisons of demographic and clinical/bacterial parameters according to the groups (control or diseased) and according to the psychosocial context as well as the evolution after SRP of clinical/bacterial parameters from periodontitis patients were carried out using Chi-2 or Fisher’s exact tests for qualitative variables, as appropriate, and Wilcoxon signed-rank test for quantitative ones. Correlations between questionnaires scores (STAI-YA, STAI-YB and PSS) and salivary cortisol were computed with Pearson’s coefficient correlation. Logistic regression models were used to measure the effect of stress and anxiety on the evolution of periodontal clinical parameters. Finally, to assess factors associated with high cortisol concentrations, linear regression model was evaluated with a stepwise approach with a significance level for entry (sle) of 0.2 and a significance level of staying (sls) of 0.05. The significance level was set at 5%. The analyses were performed using SAS software version 9.4 (SAS Institute, Inc., Cary, NC, USA).

## 3. Results

### 3.1. Demographic and Site Characteristics

Thirty control patients without periodontitis (between 22 and 70 years, median age 55.0 years) and thirty diseased patients (between 38 and 65 years, median age 51.0 years) were included in this study. No difference between groups according to age and gender (control and diseased group: 17 females (56.7%) and 13 males (43.3%)) in each group was observed (*p* = 0.91 and *p* = 1.00, respectively). The demographic data and the psychosocial characteristics of the patients included are provided in Table 1.

**Figure 1:**
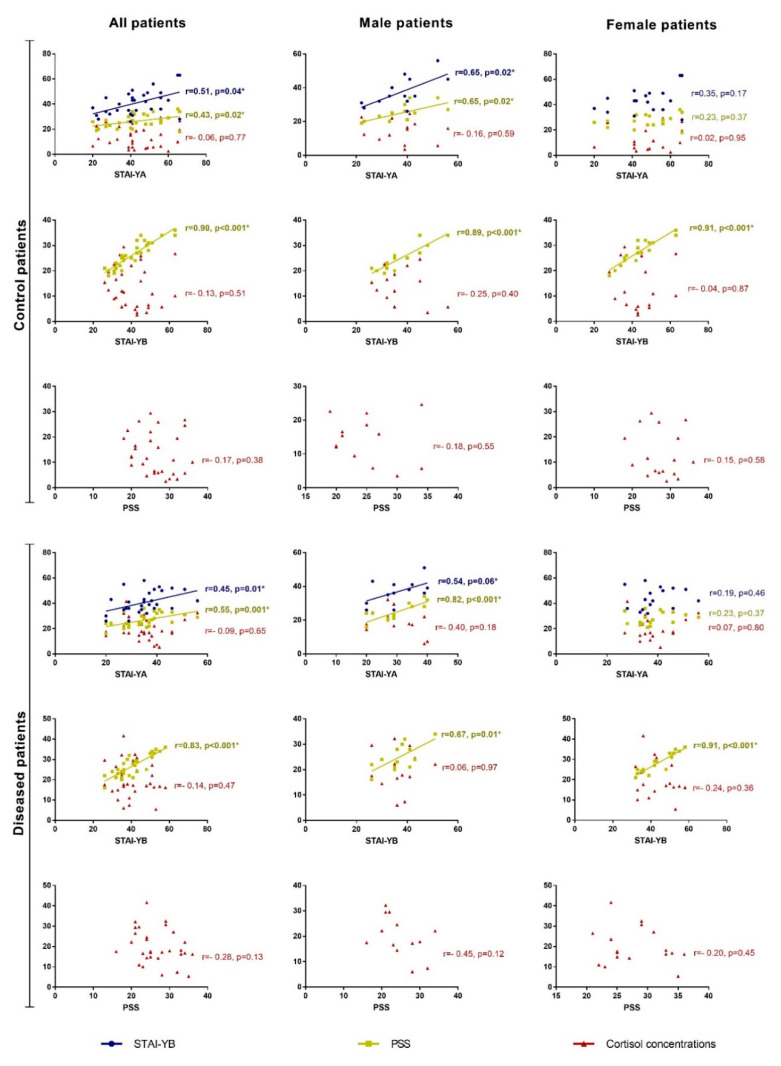
Correlations between questionnaires scores and cortisol concentrations. STAI-YA: State anxiety inventory; STAI-YB: Trait anxiety inventory; PSS: Perceived Stress Scale; r = Rho (correlation coefficient). Correlations between scores of STAI-YA, STAI-YB, PSS and cortisol concentration were analyzed among the control and diseased patients’ groups, in all patients and among men and women of each group. Pearson correlation coefficients and significance are given facing of each tested correlation. The significant correlations have been highlighted with a star.

A higher proportion of smokers was found in the diseased group (*p* = 0.02). Concerning the patients’ scores on the different questionnaires, only those of STAI-YA scale showed a significant higher score in the control group than in diseased group (*p* = 0.007) and a significantly different distribution which includes more very anxious patients in control group (*p* = 0.006) (Table 1). A statistically significant positive correlation between the STAI-YB and PSS scores was observed, regardless of gender and group (Figure 1). Regarding the cortisol levels, they were not correlated with the scores of the different stress and anxiety questionnaires for all the patients of both groups (Figure 1).

**Figure 2:**
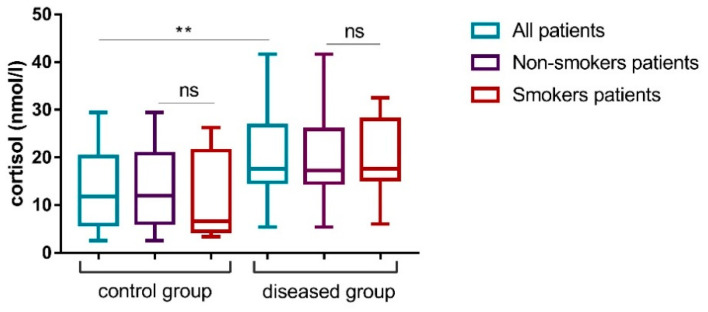
Cortisol concentrations in all patients and according to the smoking status in control and diseased group. ** *p* < 0.01; ns = non-significant.

Conversely, salivary cortisol concentrations were higher in the diseased group (*p* = 0.009) (Figure 2). An analysis of the impact of smoking status on demographic and psychosocial data showed no significant difference in cortisol concentrations or in questionnaires scores between smokers and non-smokers patients (Supplementary Table S1 and Figure 2). Nevertheless, among the anxious patient of diseased group, the proportion of smokers was higher than non-smokers (STAI-YA, *p* = 0.03) (Supplementary Table S1).

The site characteristics with clinical and bacterial data before and after SRP are provided in Supplementary Figure S1 and Table S2. None of the 30 periodontitis patients were lost to follow-up during the study and no adverse events were reported. Before SRP, significant differences between pathological and healthy sites or control sites were observed for each parameter (PlI, GI, teeth mobility, BoP, PPD, CAL) (*p* < 0.001). After SRP, comparisons of periodontal parameters still indicate significant difference between healthy and pathological sites from periodontitis patients about all parameters (*p* < 0.001). Moreover, significant improvements were found in GI, PPD and CAL in pathological sites after SRP (Supplementary Figure S1a–f, *p* < 0.001 for each parameter). Regarding bacterial detections in pathological sites, a significant reduction in the total bacterial load (Supplementary Figure S1g, *p* < 0.001) and in the frequency of bacteria detection of both red and orange complexes in the pathological sites (Supplementary Figure S1h, *P. gingivalis* (*p* = 0.03), *T. denticola* (*p* = 0.03), *T. forsythia* (*p* = 0.03), *C. rectus* (*p* = 0.008) and *E. nodatum* (*p* = 0.04)) were observed after SRP.

### 3.2. Stress/Anxiety Context and Clinical Parameters

The impact of stress and anxiety on periodontal clinical parameters in diseased group, prior to any treatment, and in control group are presented in Table 2 and Supplementary Table S3 respectively. No significant differences were observed between all clinical parameters of patients with or without anxiety, as determined by STAI-YB and PSS, in the control group. Similar results were observed in diseased patients, except for PlI scores which presented a different distribution of the categories according to anxiety (anxious patients and very anxious versus non-anxious patients) (*p* = 0.03). In the diseased group, the non-anxious patients presented a lower percentage of visible plaque on their selected teeth than anxious patients.

Clinical factors associated with high salivary cortisol concentrations in pathological sites from diseased patients before SRP were determined using multiple linear regression (Table 3). An association was observed between cortisol and PPD in bivariate (*p* = 0.047) and multivariate analysis (*p* = 0.04). No significant association was found with the other periodontal parameters.

After SRP, no association between periodontal parameters and psychosocial factors, both anxious and stress auto-questionnaires was found. (Table 4). However, when the extreme categories were compared, diseased patients, with high anxiety (according to the self-assessment of anxiety—STAIYA) seem to have a risk 5.33 times higher of having an enhancement of their supragingival biofilm than non-anxious one (0.89–31.92, *p* = 0.07) (result not shown).

### 3.3. Stress/Anxiety and Periodontal Bacteria

No linear relationship of salivary cortisol concentrations with the presence of specific periodontopathogens or with total amount of microbiota was found before SRP (Table 3). However, there may be a statistical trend of a linear association between the presence of *P. micra* and low levels of salivary cortisol in bivariate and multivariate analyses (*p* = 0.06). In addition, no correlation between salivary cortisol concentrations and the total amount of microbiota was identified (Supplementary Figure S2, *p* > 0.05).

The presence of periodontopathogens according to anxiety and stress levels before treatment was described in Table 5. In the diseased group, *T. forsythia* was significantly recovered in periodontal pockets samples of all highly stressed patients in comparison with non-highly stressed patients (*p* = 0.03), and *A. actinomycetemcomitans* was exclusively detected in pockets of non-anxious patients (*p* = 0.08). None of the other identified bacteria demonstrated a difference in their detection in relation to the stress or anxiety parameters.

Regarding the relationship between the evolution before and after SRP of the presence of some periodontopathogens bacteria according to psychosocial indicators, some significant associations were observed (Table 6). The evolution of *P. gingivalis* varied significantly according to STAI-YA categories (*p* = 0.03) with higher persistence and appearance in anxious and non-anxious patients by comparison with very-anxious patients from diseased group. No difference in the evolution of any periodontopathogens has been observed with STAI-YB categories (Supplementary Table S4). According to the PSS scale, in diseased group, significant differences in the evolution of detection of *T. forsythia*, *F. nucleatum* and *C. rectus* were observed after SRP. *T. forsythia* and *C. rectus* were more detected in very stressed patients than non-stressed or managed stress patient (*p* = 0.02, *p* = 0.009 respectively). Regarding *F. nucleatum* detection, this species was more persistent in managed stress and stressed patients than non-stressed patients. However, none of these results remained significant after grouping anxiety and stress categories: anxious and very anxious patients for STAI-YA and non-stressed and stress-managed patients for PSS (*p* > 0.05).

## 4. Discussion

Very few studies have investigated the correlation between psychosocial stress and periodontal clinical and microbiological parameters, including the detection of gram-negative bacteria in periodontal disease and the evolution of their presence after SRP. The main results of this study are: (i) a lack of correlation between cortisol concentrations and scores obtained on the stress and anxiety self-report questionnaires, (ii) a positive linear association between cortisol levels and periodontal pocket depth, and (iii) a modulation of the presence within the periodontal pocket of certain periodontopathogens, particularly those of the red Socransky complex, according to self-reported stress or anxiety level in periodontitis patients.

Stress or anxiety have been identified as risk factors of various pathologies that can influence inflammatory processes and lead to the development of inflammatory diseases [14]. Acute and chronic psychosocial stress seem to play a role in low-grade inflammation in humans and potential relationships between inflammatory response to this acute psychosocial stress and long-term development of diseases such as cardiovascular disease, diabetes mellitus or periodontal diseases could exist [33,34]. In response to acute psychosocial stress, a transient increase in systemic inflammation has been indeed observed with higher responses in people with either depression or a strong decrease in self-esteem or self-confidence [34] Atherosclerosis is a good example of a pathological process linked to stress and influenced by inflammatory molecules. Even if this disease is the consequence of a local inflammatory response, the low-grade inflammation at a systemic level of the patient, may contribute to the development of atherosclerotic plaques and stimulate the development of pre-existing plaques. Stress is a known predictor of the atherosclerotic process with a negative effect on the cardiovascular prognosis. Moreover, studies have shown that low-grade inflammation of the body can contribute to stimulate insulin resistance and thus an increased risk of type 2 diabetes, or to tumour progression [35,36,37]. Therefore, stressors seem to be involved or to be a risk factor for developing or worsening of the diseases previously mentioned, but also of periodontal diseases. Moreover, periodontitis has also been identified as being able to have adverse impact to systemic health, to influence the progression or even the initiation of these systemic diseases. The concerned pathways include bacterial translocation, the dysbiotic microbial community evading the host’s immune response while favouring its inflammatory aspects and leading to a low-grade systemic inflammation [4,38,39]. Therefore, periodontitis progression could be influenced by stress/anxiety psychosocial factors through a psychoneuroimmunological or a behavioral response [14,15]. Psychosocial factors could thus be considered as a common risk factor between systemic and periodontal conditions, with periodontal disease itself being considered as a potential risk factor for many systemic conditions.

Psychological context can be measured both subjectively, through perceived stress and/or anxiety levels, and objectively via cortisol level. In this study, the STAI-YB and the Cohen Perceived Stress Scale (PSS) questionnaires were highly correlated which strengthens the reliability of detected psychosocial stressors. Psychosocial context could influence the onset and development of periodontitis through changes in patient behaviour and altered biological responses [40]. Considering behaviour, this is consistent with our results showing that anxious and very anxious patients from the diseased group had the lowest oral hygiene. Poor oral hygiene has been identified as a factor inducing a major ecological pressure and influencing the shift from symbiotic to dysbiotic microbiome. In dysbiosis, the finely tuned balance of the different components of the oral ecosystem is disturbed, allowing pathogenic bacteria to express their virulence factors [41]. Through the evaluation of the hypothalamus–pituitary–adrenal cortex axis, the salivary cortisol level is frequently used as a biological marker of psychological stress. In our study, salivary cortisol concentrations were significantly higher in the periodontitis group than in the control group, and were correlated with increased periodontal pocket depth, both results being consistent with previous studies [42,43]. Glucocorticoid production could result in the production of pro-inflammatory cytokines participating to periodontal degradation [44,45,46]. This overproduction of cytokines might be one mechanism that links inflammation, cortisol and the bacterial environment, and needs to be clarified. However, no correlation between cortisol concentrations and both stress and anxiety questionnaires has been established. The lack of correlation could be explained by the way individuals manage their stress. The scores and categories of STAI-YB and PSS were not significantly different between diseased and control groups. These results are similar to those of Patacchioli et al. (2001) [47] demonstrating that patients with inflammatory bowel syndrome or a chronic inflammatory pathology such as periodontal disease, did not differ from the control group in their psychological dimensions investigated by the STAI and PSS questionnaires. Our results also confirm that the psychological context can only be considered as a risk factor and not an inducing factor of periodontitis.

Moreover, in our study, all bacteria significantly associated with stress were gram negative and could to some extent potentially participate in the patient’s psychosocial disorders. Indeed, some authors have shown that the administration of pro-inflammatory cytokines and lipopolysaccharide can trigger depressive and/or anxiety-like behaviour in animal models [48,49,50]. Furthermore, other authors have shown that stress could modulate the composition of microbiota and that the growth of several anaerobes could be modified by stress hormones delivered in the saliva, cortisone as well as catecholamines [16,51]. Our study revealed a significant correlation between certain periodontopathogens and psychosocial stressors and differences in detection evolution according to stress or anxiety context such as for *P. gingivalis* and *T. forsythia*, red complex bacteria. Before SRP, statistical analysis showed a quite significant association between cortisol concentration and *P. micra*. Hormones such as adrenaline and noradrenaline could also induce modulations of bacterial growth. Indeed, a significant enhancement for *P. micra* growth and an inhibitory effect for *A. actinomycetemcomitans* and some bacteria of the red and orange complexes were described [18,52]. A recent study based on a metatranscriptomic approach showed that glucocorticoids can directly induce changes in gene expression profiles of the oral microbiome, similar to those observed in periodontitis patients [53]. Moreover, patients suffering from periodontitis with a high-stress level exhibited a significant correlation between cortisol levels and *T. forsythia* detection compared to patients with low-stress levels or stress-managed patients. This result is consistent with changes observed in salivary microbiota of healthy patients subjected to academic-related stress [54]. The interactions between psychological stress and the development of infectious disease have recently been investigated in the concept of “microbial endocrinology” but the mechanisms by which stress-related molecules interact with microbiota and modulate the metabolism, the virulence and growth profile of bacteria are not clearly understood and regarding periodontopathogens are little evaluated [55,56,57]. Some bacterial receptors, such as QseC in *E. coli* and QseBC in *A. actinomycetemcomitans* could be activated by catecholamines initiating the bacterial growth [58]. The significant correlations between bacteria and stress categories varied before and after SRP. This observation could be explained by variation in the subgingival biofilm composition inducing different bacterial synergism, but also by species-dependent effects of stress hormone on certain anaerobes and within a same genus as described by Robert et al. (2002) for *Actinomyces* spp. [18]. Moreover, the stress hormone released into the oral cavity did not only influence the growth of some periodontal bacteria but also could increase their virulence by enhancing the expression of genes related to oxidative stress, iron acquisition and haemolytic activity, as described for *P*. gingivalis [59]. The ability of periodontopathogens to potentially influence human behavior through their capacity to produce and recognize neurochemicals, as described with *Campylobacter jejuni* in mice model, have to be explored [60].

This study describes preliminary results based on a cohort of 30 individuals in each group. Some limitations in this clinical study can be mentioned: (i) the analysis of periodontopathogens presence and stress/anxiety levels in groups and subgroups of limited size, and (ii) the detected bacterial species limited to certain periodontopathogens widely considered as the most virulent one which explore only a tiny part of the periodontal pocket microbiota, (iii) the quantification of other molecules such as neuroendocrine hormones to evaluate the level of stress and anxiety of the patient. A larger scale, multicenter and targeted study which would include patients suffering from severe periodontitis (stage III and IV) and being highly stressed or anxious could in the future allow a better understanding of the interrelationships between psychosocial factors (stress/anxiety) microbiota within sub-gingival biofilms and periodontal degradation.

## 5. Conclusions

Our data indicate that stress and anxiety in diseased patients appear to be associated with different microbial colonization or persistence after SRP of Socransky’s red or orange complex bacteria between anxious personality/highly stressed and non-anxious/stressed patients. However, salivary cortisol concentrations did not appear to be correlated with self-report scores of stress/anxiety, nor with bacterial detection or total bacterial load; but only with periodontal degradation (PPD). Interrelations between bacterial environment and psychological context of patients seem to occur and require further research for better understanding. The development of interventions to reduce the behavioural and biological sequelae of psychological stress or anxiety should be an integral part of the periodontal treatment plan.

## Figures and Tables

**Table 1:** Demographic and psychosocial characteristics of the study population.

Demographic and Psychosocial Characteristics			Control Group (*n* = 30)			Diseased Group (*n* = 30)			*p*-Value *
			*N*	%/Med	[Min-Max]	*N*	%/Med	[Min-Max]	
**Demographic Information**									
**Gender**	Male		13	43.3		17	43.3		1.00
	Female		17	56.7		13	56.7		
**Age**			30	55.0	[22.0–70.0]	30	51.0	[38.0–65.0]	0.91
**Smoking Status**	Non-smokers		25	83.3		17	56.7		**0.02**
	Smokers		5	16.7		13	43.3		
**Psychosocial Characteristics**									
**Anxiety**	STAI-YA (scores)		30	41.0	[20.0–66.0]	30	35	[20.0–56.0]	**0.007**
	STAI-YA (classes)	Non-anxious (men < 37 and women < 42)	12	40.0		23	76.7		**0.006**
		Anxious	10	33.3		6	20.0		
		Very anxious (men >48 and women >55)	8	26.7		1	3.3		
	STAI-YB (scores)		30	41.0	[26.0–63.0]	30	39.0	[26.0–58.0]	1.00
	STAI-YB (classes)	Non-anxious (men <39 and women <47)	19	63.3		19	63.3		0.26
		Anxious	8	26.7		11	36.7		
		Very anxious (men >51 and women >61)	3	10.0		0	0.0		
**Stress**	PSS (scores)		30	26.0	[18.0–36.0]	30	25.0	[16.0–36.0]	0.85
	PSS (classes)	Non-stressed (<21)	5	16.6		2	6.6		0.51
		Managed stress	11	36.7		14	46.7		
		Very stressed (>27)	14	46.7		14	46.7		

Notes: Med: median; [Min-Max]: [Minimum-Maximum]; STAI-YA: State anxiety inventory; STAI-YB: Trait anxiety inventory; PSS: Perceived Stress Scale. * Chi-2 or Fisher’s exact test was used for qualitative variables and the Wilcoxon test for quantitative ones. Significant values are shown in bold.

**Table 2:** Periodontal clinical parameters according to the psychosocial context of diseased patients.

Periodontal Clinical Parameters	STAI-YB								PSS						
		Non-Anxious			Anxious and Very Anxious				Non-Highly Stressed			Highly Stressed			
	Scores	*N*	%/Med	[Min-Max]	*N*	%/Med	[Min-Max]	*p*-Value *	*N*	%/Med	[Min-Max]	*N*	%/Med	[Min-Max]	*p*-Value *
**Diseased patients (healthy + pathological sites)**															
**PlI (scores)**	0	25	43.9		18	54.5		**0.03**	22	45.8		21	50		0.17
	1	22	38.6		4	12.1			18	37.5		8	19		
	2	9	15.8		9	27.3			7	14.6		11	26.2		
	3	1	1.8		2	6.1			1	2.1		2	4.8		
**GI (scores)**	0	10	17.5		3	9.1		0.51	8	16.7		5	11.9		0.88
	1	26	45.6		15	45.5			21	43.8		20	47.6		
	2	17	29.8		10	30.3			15	31.3		12	28.6		
	3	4	7.0		5	15.2			4	8.3		5	11.9		
**PPD (mm)**		57	6.0	[1.0–11.0]	33	6.0	[1.0–11.0]	0.73	48	6.5	[1.0–10.0]	42	6.0	[1.0–11.0]	0.64
**CAL (mm)**		57	7.0	[1.0–11.0]	33	7.0	[1.0–16.0]	0.53	48	7.0	[1.0–16.0]	42	6.0	[1.0–11.0]	0.84
**Teeth mobility**		21	36.8		13	39.4		0.81	17	35.4		17	40.5		0.62

Notes: Med: median; [Min-Max]: [Minimum-Maximum]; STAI-YB: Trait anxiety inventory; PSS: Perceived Stress Scale; PlI: Plaque Index; GI: Gingival Index; PPD: Periodontal pocket depth; CAL: Clinical attachment level. * Chi-2 or Fisher’s exact test was used for qualitative variables and Wilcoxon test for quantitative ones. Significant values are shown in bold.

**Table 3:** Associated factors with high levels of salivary cortisol in pathological sites from diseased patients.

Associated Factors		Salivary Cortisol (mean)	*p*-Value Bivariate Regression Analysis	Beta	*p*-Value Multivariate Regression Analysis
Smoking status	No	19.9	0.95		-
	Yes	20.0			
PPD (mm)	5–6	16.3	**0.047**	−6.1	**0.04**
	7–8	22.4		0.0	
	≥9	20.5		−2.5	
PlI (mean)	0	19.7	0.96		-
	1	19.8			
	2	19.9			
	3	22.6			
GI (mean)	1	20.0	0.96		-
	2	19.6			
	3	20.6			
*A. actinomycetemcomitans*	No	19.9	0.94		-
	Yes	20.2			
*P. gingivalis*	No	22.6	0.50		-
	Yes	18.8			
*P. intermedia*	No	19.2	0.33		-
	Yes	21.4			
*T. forsythia*	No	21.0	0.76		-
	Yes	19.8			
*T. denticola*	No	20.5	0.79		-
	Yes	19.8			
*P. micra*	No	23.1	0.06	4.3	0.06
	Yes	18.6		0.0	
*F. nucleatum*	No	23.6	0.53		-
	Yes	19.9			
*C. rectus*	No	22.8	0.14	4.1	0.10
	Yes	19.0		0.0	
*E. nodatum*	No	20.2	0.78		-
	Yes	19.6			
*E. corrodens*	No	18.3	0.26		-
	Yes	21.3			
*Capnocytophaga* spp.	No	20.7	0.54		-
	Yes	19.3			

*Notes*: PlI: Plaque Index; GI: Gingival Index; PPD: Periodontal pocket depth; No/Yes: Absence of detection/Detection of the targeted periodontopathogen in the GCF sample. One-factor variable analysis test if equality of variances, Kruskal-Wallis test if not for qualitative variables, test resulting from a simple linear regression for quantitative variables. Relevant individual factors were identified as those which were found to be associated in the univariate analyses at a 20% threshold. (*n* = 60. R^2^ = 0.2). Significant values are shown in bold.

**Table 4:** Effect of stress and anxiety on the evolution before-after treatment of periodontal clinical parameters from diseased patients.

Periodontal Clinical Parameters’ Variations		Anxious/Very Anxious Versus Non-Anxious Patients					Very Stressed Versus Non-Stressed/Managed Stress Patients			
		STAI-YA			STAI-YB			PSS		
		OR	95%CI	*p*-Value	OR	95% CI	*p*-Value	OR	95%CI	*p*-Value
PlI	Improvement	1.43	0.35–5.83	0.62	0.34	0.10–1.13	0.08	0.95	0.31–2.85	0.92
	Degradation	4.00	0.73–21.84	0.11	1.48	0.32–6.89	0.62	1.48	0.32–6.90	0.62
GI	Improvement	1.05	0.10–10.70	0.97	1.01	0.13–7.82	0.99	0.89	0.12–6.50	0.90
	Degradation	na	na	na	na	na	na	na	na	na
PPD	Decrease	0.38	0.07–1.94	0.24	0.69	0.14–3.43	0.65	0.64	0.13–3.16	0.58
	Increase	0.37	0.073–1.94	0.99	2.67	0.16–45.14	0.50	0.38	0.02–6.35	0.50
BOP	Disappearance	2.03	0.5–7.42	0.29	2.59	0.77–9.66	0.12	2.82	0.84–9.51	0.09
	Emergence	0.41	0.04–3.83	0.43	0.99	0.21–4.61	0.98	1.03	0.24–4.35	0.97
Teeth Mobility	Disappearance	8.20	0.67–99.70	0.10	3.37	0.29–39.69	0.33	2.64	0.22–30.97	0.44
	Emergence	2.05	0.33–12.82	0.44	0.34	0.04–3.10	0.34	2.64	0.44–15.72	0.29

*Notes*: Improvement/decrease or degradation/increase of each periodontal parameters have been detailed according to the different categories of STAI-YA, STAI-YB and PSS scales. na = not applicable (no degradation of GI). Odd ratio (OR) were obtained by logistic regression.

**Table 5:** Bacterial environment according to the psychosocial context in pathological samples from diseased patients before periodontal treatment.

Periodontopathogens	STAI-YB					PSS				
	Non-Anxious		Anxious and Very Anxious			Non-Highly Stressed			Highly Stressed	
	*N* = 38	%	*N* = 22	%	*p*-Value *	*N* = 32	%	*N* = 28	%	*p*-Value *
*A. actinomycetemcomitans*	*6*	*15.8*	*0*	*0.0*	0.08	2	6.3	4	14.3	0.40
*P. gingivalis*	24	63.2	18	81.8	0.13	22	68.8	20	71.4	0.82
*P. intermedia*	11	28.9	9	40.9	0.34	9	28.1	11	39.3	0.36
*T. forsythia*	34	89.5	20	90.9	1.00	26	81.3	28	100.0	**0.03**
*T. denticola*	31	81.6	17	77.3	0.69	27	84.4	21	75.0	0.37
*P. micra*	25	65.8	17	77.3	0.35	22	68.8	20	71.4	0.82
*F. nucleatum*	37	97.6	22	100.0	1.00	31	96.9	28	100.0	1.00
*C. rectus*	29	76.3	17	77.3	0.93	27	84.4	19	67.9	0.13
*E. nodatum*	20	52.6	9	40.9	0.38	18	56.3	11	39.3	0.19
*E. corrodens*	16	42.1	12	54.5	0.35	14	43.8	14	50.0	0.63
*Capnocytophaga* spp.	20	52.6	13	59.1	0.63	18	56.3	15	53.6	0.84

*Notes*: STAI-YB: State Trait Anxiety Inventory-Trait anxiety; PSS: Perceived Stress Scale. * Chi-2 or Fisher’s exact test were applied. Significant values are shown in bold.

**Table 6:** Effect of stress and anxiety on the detection of some periodontal bacteria before and after SRP treatment from diseased patients.

Periodontopathogens’ Variations	STAI-YA							STAI-YB					PSS						
	Non-Anxious		Anxious		Very Anxious			Non-Anxious		Anxious			Non-Stressed		Managed Stress		Very Stressed		
	*N*	%	*N*	%	*N*	%	*p*-Value *	*N*	%	*N*	%	*p*-Value *	*N*	%	*N*	%	*N*	%	*p*-Value *
***P. gingivalis***																			
Never detected	14	30.4	2	16.7	0	0.0	**0.03**	12	31.6	2	18.2	0.09	0	0.0	8	28.6	8	28.6	0.14
Disapperance	9	19.6	0	0.0	2	100		9	23.7	2	9.1		3	75	4	14.3	4	14.3	
Appearance	2	4.3	0	0.0	0	0.0		2	5.3	0	0.0		0	0.0	2	7.1	0	0.0	
Always detected	21	45.7	10	83.3	0	0.0		21	39.5	16	72.7		1	25	14	50	16	57.1	
***T. forsythia***																			
Never detected	5	10.9	0	0.0	0	0.0	0.86	3	7.9	2	9.1	1.00	2	50	3	10.7	0	0.0	**0.02**
Disappearance	6	13	1	8.3	0	0.0		5	13.2	2	9.1		1	25	3	10.7	3	10.7	
Appearance	1	2.2	0	0.0	0	0.0		1	2.6	0	0.0		0	0.0	1	3.6	0	0.0	
Always detected	34	73.9	11	91.7	2	100		29	76.3	18	81.8		1	25	21	75	25	89.3	
***F. nucleatum***																			
Never detected	0	0.0	0	0.0	0	0.0	0.22	0	0.0	0	0.0	0.77	0	0.0	0	0.0	0	0.0	**0.049**
Disappearance	4	8.7	0	0.0	1	50		4	10.5	1	7.1		2	50	1	3.6	2	7.1	
Appearance	1	2.2	0	0.0	0	0.0		1	2.6	0	0.0		0	0.0	1	3.6	0	0.0	
Always detected	41	89.1	12	100	1	50		33	86.8	13	92.9		2	50	26	92.9	26	92.9	
***C. rectus***																			
Never detected	8	17.4	1	8.3	2	100	0.15	7	18.4	4	18.2	1.00	2	50	1	3.6	8	28.6	**0.01**
Disappearance	14	30.4	2	16.7	0	0.0		10	26.3	6	27.3		2	50	10	35.7	4	14.3	
Appearance	2	4.3	1	8.3	0	0.0		2	5.3	1	4.5		0	0.0	2	7.1	1	3.6	
Always detected	22	47.8	8	66.7	0	0.0		19	50.0	11	50.0		0	0.0	15	53.6	15	53.6	

Notes: STAI-YA: State-Trait Anxiety Inventory—State anxiety; STAI-YB: State Trait Anxiety Inventory-Trait anxiety (two classes because no periodontitis patients were very anxious); PSS: Perceived Stress Scale; Only significant results are shown here. The full table is presented in the supplementary data (Table S4). * Fisher’s exact test was used. Significant values are shown in bold.

## Supplementary Materials

The following are available online at https://www.mdpi.com/1660-4601/17/20/7651/s1, The following are provided in supplementary data files: Table S1: Demographic and psychosocial data according to the smoking status, Figure S1: Clinical and bacterial findings in selected sites before and after SRP, Table S2: Periodontal status according to the collected sites from control and periodontitis groups; Table S3: Periodontal clinical parameters according to the psychosocial context of control patients, Figure S2: Correlation between total microbiota count from pathological sites and cortisol concentrations in diseased patients, Table S4: Effect of stress and anxiety on the detection of periodontal bacteria before and after SRP treatment from diseased patients.
