# Supplementary material for: Relations of Psychosocial Factors and Cortisol with Periodontal and Bacterial Parameters: A Prospective Clinical Study in 30 Patients with Periodontitis Before and After Non-Surgical Treatment
Source: Int J Environ Res Public Health. 2020 Oct 20;17(20):7651. doi: 10.3390/ijerph17207651 (PMC7588876; doi:10.3390/ijerph17207651)
Supplement: Supplementary file 1 [file ijerph-17-07651-s001.pdf]

Supplementary data

Relations of psychosocial factors and cortisol with periodontal and bacterial parameters: A prospective clinical study in 30 patients with periodontitis before and after non-surgical treatment

Marie Dubar, Isabelle Clerc-Urmès, Cédric Baumann, Céline Clément, Corentine Alauzet, Catherine Bisson.

Table S1 – Demographic and psychosocial data according to the smoking status

			**Control group (n=30)**							**Diseased group (n=30)**						
			**Non-smokers**			**Smokers (n=5)**				**Non-smokers**			**Smokers (n=13)**			
Demographic and psychosocial			**N**	**% / Med**	**[Min-Max]**	**N**	**% / Med**	**[Min-Max]**	***p-Value****	**N**	**% / Med**	**[Min-Max]**	**N**	**% / Med**	**[Min-Max]**	***p-Value****
**Demographic information**																
**Gender**	Male		11	44.0		2	40.0		0.19	7	35.3		6	53.9		0.40
	Female		14	56.0		3	60.0			11	64.7		6	46.1		
**Age**			30	55.0	[22.0-70.0]	30	55.0	[49.0-58.0]	0.67	30	60.0	[40.0-65.0]	30	47.0	[38.0-64.0]	0.07
**Psychosocial characteristics**																
**Anxiety**	STAI-YA		30	41.0	[22.0-66.0]	30	40.0	[20.0-52.0]	0.19	30	34.0	[22.0-51.0]	30	36.0	[20.0-56.0]	0.76
	STAI-YA (classes)	N-A	9	36.0		3	60.0		0.24	16	94.1		7	53.8		**0.03**
		A +	16	64.0		2	40.0			1	5.9		6	46.2		
	STAI-YB		30	42.0	[26.0-63.0]	30	37.0	[32.0-56.0]	0.30	30	39.0	[26.0-58.0]		39.0	[26.0-55.0]	0.79
	STAI-YB (classes)	N-A	15	60.0		4	80.0		0.30	10	58.8		9	69.2		0.07
		A +	10	40.0		1	20.0			7	41.2		4	30.8		
**Stress**	PSS (scores)		30	26.0	[18.0-36.0]	30	26.0	[21.0-34.0]	0.77	30	25.0	[21.0-36.0]	30	28.0	[16.0-34.0]	0.98
	PSS (classes)	N-S +	13	52.0		3	60.0		0.95	10	58.8		6	46.2		0.45
		V-	12	48.0		2	40.0			7	41.2		7	53.8		

*Notes: Med: median; [Min-Max]: [Minimum-Maximum]; STAI-YA = State anxiety inventory; STAI-YB = Trait anxiety inventory; PSS = Perceived Stress Scale; N-A= non-anxious; A= anxious; V-A= very anxious; N-S= non-stressed; M-S= managed stress; V-S= very-stressed. *Chi-2 or Fisher's exact test was used for qualitative variables and Wilcoxon test for quantitative ones.* Significant values are shown in bold.

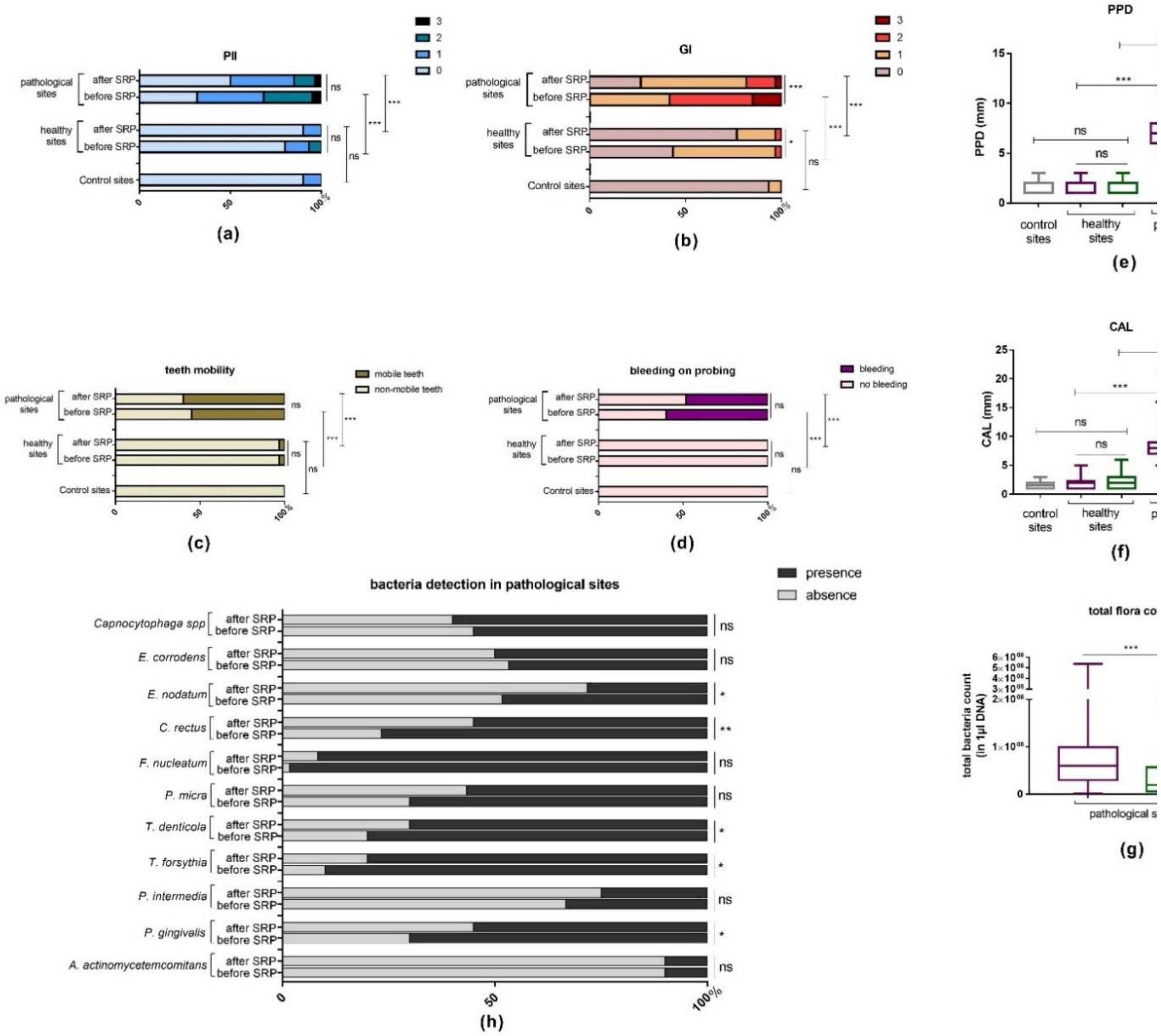

Figure 1. – Clinical and bacterial findings in selected sites before and after SRP.

(**a**) Plaque Index - PlI; (**b**) Gingival Index - GI; (**c**) Teeth mobility; (**d**) Bleeding on probing - BoP; (**e**) Periodontal pocket depth - PPD; (**f**) Clinical attachment level - CAL; (**g**) Total flora count and (**h**) bacterial detection frequencies. Chi-2 test was used for qualitative variables and Wilcoxon test for quantitative ones. ***p<0.001, **p<0.01, *p<0.05, ns=non-significant.

<u>Table S2</u> – Periodontal status according to the collected sites from control and periodontitis groups.

		**Control group (n=30)**	**Periodontitis group (n=30)**							
**Clinical parameters**		**Control sites (n=30)**	**Healthy sites (n=30)**				**Pathological sites (n=60)**			
	**classes**		**BEFORE SRP**	**AFTER SRP**	***p-value**** [a]	***p-value**** [b]	**BEFORE SRP**	**AFTER SRP**	***p-value**** [c]	***p-value**** [d]
**PlI score (%)**	0	90.0	80.0	90.0	0.40	1.00	31.7	50	0.111	**<0.001**
	1	10.0	13.3	10.0			36.7	35		
	2	0.0	6.7	0.0			26.7	11.7		
	3	0.0	0.0	0.0			5.0	3.3		
**GI score (%)**	0	93.3	43.3	76.7	**0.02**	0.07	0.0	26.7	**<0.001**	**<0.001**
	1	6.7	53.3	20.0			41.7	55.0		
	2	0.0	3.3	3.3			43.3	15.0		
	3	0.0	0.0	0.0			15.0	3.3		
**Mobility (%)**		0.0	3.3	3.3	1.00	0.31	55	60	0.317	**<0.001**
**BOP (%)**		0.0	0.0	0.0	1.00	1.00	60	48.3	0.275	**<0.001**
**PPD (Med [Min-Max])**		1 [1-3]	2 [1-3]	1 [1-3]	0.42	1.00	7 [5-11]	5 [2-13]	**<0.001**	**<0.001**
**PPD classes (%)**	<4mm	100.0	100.0	100.0	1.00	1.00	0.0	10	**<0.001**	**<0.001**
	4-6 mm	0.0	0.0	0.0			33.3	66.7		
	7-8mm	0.0	0.0	0.0			43.3	18.3		
	>8mm	0.0	0.0	0.0			23.3	5.0		
**CAL (Med [Min-Max])**		1.5 [1-3]	2 [1-5]	2 [1-6]	0.95	0.09	8 [5-16]	6 [3-20]	**<0.001**	**<0.001**
**CAL classes (%)**	<4mm	100.0	100.0	100.0	1.00	1.00	0.0	5.0	**<0.001**	**<0.001**
	4-6mm	0.0	0.0	0.0			21.7	53.3		
	7-8mm	0.0	0.0	0.0			36.7	26.7		
	>8mm	0.0	0.0	0.0			41.7	15.0		

Notes: Med: median;[Min-Max]: [Minimum-Maximum]; PlI: Plaque Index; GI: Gingival Index; Mobility: mobile teeth; BOP: Bleeding on probing; PPD: Periodontal pocket depth; CAL: Clinical attachment level; SRP: scaling and root planing – periodontal treatment; a: comparison between healthy sites before and after periodontal treatment; b: comparison between control and healthy sites after periodontal treatment; c: comparison between pathological sites before and after periodontal treatment; d: comparison between healthy and pathological sites after periodontal treatment;.**Chi-2 or Fisher test was used for qualitative variables and Wilcoxon test for quantitative ones.* Significant values are shown in bold.

<u>Table S3</u> - Periodontal clinical parameters according to the psychosocial context of control patients.

		STAI-YB							PSS						
		**Non-anxious**			**Anxious and very anxious**				**Non-highly stressed**			**Highly stressed**			
**Periodontal clinical parameters**		**N**	**% / Med**	**[Min-Max]**	**N**	**% / Med**	**[Min-Max]**	***p-Value****	**N**	**% / Med**	**[Min-Max]**	**N**	**% / Med**	**[Min-Max]**	***p-Value****
**Control patients (control site)**															
**PlI scores**	0	17	89.5		10	90.9		1.00	14	87.5		13	92.9		1.00
	1	2	10.5		1	9.1			2	12.5		1	7.1		
**GI scores**	0	17	89.5		11	100		0.52	14	87.5		14	100		0.49
	1	2	10.5		0	0			2	12.5		0	0		
**PPD (mm)**		19	1.0	[1.0-[illegible]	11	1.0	[1.0-[illegible]	0.98	16	1.5	[1.0-[illegible]	14	1.0	[1.0-[illegible]	0.40
**CAL (mm)**		19	1.5	[1.0-3.0]	11	2.0	[1.0-2.0]	0.96	16	2.0	[1.0-3.0]	14	1.0	[1.0-2.0]	0.29

Notes: Med: median;[Min-Max]: [Minimum-Maximum]; STAI-YB: Trait anxiety inventory; PSS: Perceived Stress Scale; lI: Plaque Index; GI: Gingival Index; PPD: Periodontal pocket depth; CAL: Clinical attachment level; *Chi-2 or Fisher test was used for qualitative variables and Wilcoxon test for quantitative ones.

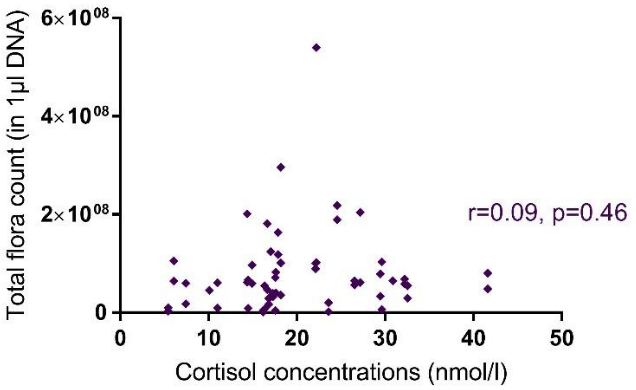

<u>Figure S2</u> – Correlation between total microbiota count from pathological sites and cortisol concentrations in diseased patients. r: Rho (correlation coefficient). Pearson correlation coefficient was used.

Table S4 - Effect of stress and anxiety on the detection of some periodontal bacteria before and after SRP treatment from diseased patients.

	STAI-YA							STAI-YB					PSS						
	Non-anxious		Anxious		Very anxious			Non-anxious		anxious			Non-stressed		Managed stress		Very stressed		
	N	%	N	%	N	%	*p-Value**	N	%	N	%	*p-Value**	N	%	N	%	N	%	*p-Value**
A. actinomycetemcomitans																			
Never detected	42	91.3	10	83.3	2	100		32	84.2	22	100.0		4	100	26	92.9	24	85.7	
Always detected	4	8.7	2	16.7	0.0	0.0	0.67	6	15.8	0	0.0	0.08	0	0.0	2	7.1	4	14.3	0.79
P. gingivalis																			
Never detected	14	30.4	2	16.7	0	0.0		12	31.6	4	18.2		0	0.0	8	28.6	8	28.6	
Disapperance	9	19.6	0	0.0	2	100		9	23.7	2	9.1		3	75	4	14.3	4	14.3	
appearance	2	4.3	0	0.0	0	0.0	**0.03**	2	5.3	0	0.0	0.09	0	0.0	2	7.1	0	0.0	0.14
Always detected	21	45.7	10	83.3	0	0.0		15	39.5	16	72.7		1	25	14	50	16	57.1	
P. intermedia																			
Never detected	30	65.2	7	58.3	0	0.0		25	65.8	12	54.5		2	50	19	67.9	16	57.1	
Disappearance	5	10.9	1	8.3	2	100		6	15.8	2	9.1		1	25	4	14.3	3	10.7	
Appearance	2	4.3	1	8.3	0	0.0	0.13	2	5.3	1	4.5	0.35	0	0.0	2	7.1	1	3.6	0.58
Always detected	9	19.6	3	25	0	0.0		5	13.2	7	31.8		1	25	3	10.7	8	28.6	
T. forsythia																			
Never detected	5	10.9	0	0.0	0	0.0		3	7.9	2	9.1		2	50	3	10.7	0	0.0	
Dsappearance	6	13	1	8.3	0	0.0		5	13.2	2	9.1		1	25	3	10.7	3	10.7	
Appearance	1	2.2	0	0.0	0	0.0	0.86	1	2.6	0	0.0	1.00	0	0.0	1	3.6	0	0.0	**0.02**
Always detected	34	73.9	11	91.7	2	100		29	76.3	18	81.8		1	25	21	75	25	89.3	
T. denticola																			
Never detected	10	21.7	0	0.0	0	0.0		6	15.8	4	18.2		2	50	2	7.1	6	21.7	
Disappearance	6	13	2	16.7	0	0.0		5	13.2	3	13.6		1	25	4	14.3	3	10.7	
Appearance	2	4.3	0	0.0	0	0.0	0.48	1	2.6	1	4.5	1.00	0	0.0	1	3.6	1	3.6	0.20
Always detected	28	60.9	10	83.3	2	100		26	68.4	14	63.6		1	25	21	75	18	64.3	
P. micra																			
Never detected	6	13	3	25	2	100		8	21.1	3	13.6		0	0.0	7	25	4	14.3	
Disappearance	14	30.5	1	8.3	0	0.0		11	28.9	4	18.2		2	50	9	32.1	4	14.3	
Appearance	6	13	1	8.3	0	0.0	0.10	5	13.2	2	9.1	0.44	0	0.0	3	10.7	4	14.3	0.31
Always detected	20	43.5	7	58.4	0	0.0		14	36.8	13	59.1		2	50	9	32.1	16	57.1	
F. nucleatum																			
Never detected	0	0.0	0	0.0	0	0.0		0	0.0	0	0.0		0	0.0	0	0.0	0	0.0	
Disappearance	4	8.7	0	0.0	1	50.0		4	10.5	1	7.1		2	50	1	3.6	2	7.1	
Appearance	1	2.2	0	0.0	0	0.0	0.22	1	2.6	0	0.0	0.77	0	0.0	1	3.6	0	0.0	**0.049**
Always detected	41	89.1	12	100	1	50		33	86.8	13	92.9		2	50	26	92.9	26	92.9	
C. rectus																			
Never detected	8	17.4	1	8.3	2	100		7	18.4	4	18.2		2	50	1	3.6	8	28.6	
Disappearance	14	30.4	2	16.7	0	0.0		10	26.3	6	27.3		2	50	10	35.7	4	14.3	
Appearance	2	4.3	1	8.3	0	0.0	0.15	2	5.3	1	4.5	1.00	0	0.0	2	7.1	1	3.6	**0.01**
Always detected	22	47.8	8	66.7	0	0.0		19	50.0	11	50.0		0	0.0	15	53.6	15	53.6	

E. nodatum																		
Never detected	22	47.8	5	41.7	2	100		18	47.4	11	50.0		1	25	13	46.4	15	53.6	
Disappearance	11	23.9	3	25	0	0.0		10	26.3	4	18.2		2	50	7	25	5	17.9	
Appearance	1	2.2	1	8.3	0	0.0	0.78	0	0.0	2	9.1	0.34	0	0.0	0	0.0	2	7.1	0.57
Always detected	12	26.1	3	25	0	0.0		10	26.3	5	22.7		1	25	8	28.6	6	21.4	
E. corrodens																			
Never detected	14	30.4	4	33.3	0	0.0		13	34.2	5	22.7		1	25	7	25	10	35.7	
Disappearance	9	19.6	1	8.3	2	100		8	21.1	4	18.2		0	0.0	7	25	5	17.9	
Appearance	12	26.1	2	16.7	0	0.0	0.27	9	23.7	5	22.7	0.62	2	50	8	28.6	4	14.3	0.58
Always detected	11	23.9	5	41.7	0	0.0		8	21.1	8	36.4		1	25	6	21.4	9	32.1	
***Capnocytophaga* spp**																			
Never detected	11	23.9	5	41.6	1	50		11	28.9	6	27.3		1	25	9	32.1	7	25	
Disappearance	5	10.9	2	16.7	0	0.0		4	10.5	3	13.6		0	0.0	2	7.1	5	17.9	
Appearance	7	15.2	2	16.7	1	50	0.32	7	18.4	3	13.6	0.96	0	0.0	4	14.3	6	21.4	0.73
Always detected	23	50	3	25	0	0.0		16	42.1	10	45.5		3	75	13	46.7	10	35.7	

Notes: STAI-YA: State-Trait Anxiety Inventory – State anxiety; STAI-YB: State Trait Anxiety Inventory-Trait anxiety (two classes because no periodontitis patients were very anxious); PSS: Perceived Stress Scale; *Fisher's exact test was used. Significant values are shown in bold.
